# On-demand peptide therapeutics for multi-year space exploration: analysis of clinical and operational relevance and recombinant production feasibility

**DOI:** 10.1038/s41526-026-00630-z

**Published:** 2026-07-09

**Authors:** John J. Donovan, Isaac Ericson, Andrew Wenthe, Katalin Kovacs, Jonas Emsley, Philip M. Williams

**Affiliations:** 1 School of Pharmacy, University of Nottingham, Nottingham, UK; 2 David Geffen School of Medicine, University of California Los Angeles, Los Angeles, CA USA; 3 Stanford University School of Medicine, Stanford, CA USA

**Keywords:** Computational biology and bioinformatics, Drug discovery, Medical research

## Abstract

Future crewed expeditions beyond Earth will span multiple years, requiring a transition away from Earth-dependent pharmaceutical supply chains. Medications degrade more rapidly in space due to radiation exposure and storage constraints, while transporting large quantities of temperature-controlled biologics is logistically impractical. These challenges necessitate the development of self-sufficient, in-situ medical capabilities for deep space missions. This study evaluates the operational and clinical relevance (OCR) and recombinant production feasibility (RPF) of peptide therapeutics for on-demand manufacturing during long-duration spaceflight. Building on established astropharmacy databases and literature, 26 peptide-based medications relevant to spaceflight were identified and ranked using ten predefined OCR and RPF criteria. OCR criteria included regulatory status, shelf stability, storage requirements, NASA Human Research Roadmap risk/impact score, and purification requirements. RPF criteria included amino acid chain length, prior recombinant production, functional assay availability, dosing requirements, and post-translational modification complexity. All criteria were scored from 0-2 points. Teriparatide achieved the highest overall score (17/20), followed by abaloparatide and amylin (16/20). Several therapeutics, including angiotensin II, daptomycin, GLP-1 agonists, G-CSF, GM-CSF, and salmon calcitonin, also demonstrated potential (14/20). This study identifies peptide therapeutics as promising candidates for in-situ production and provides a structured framework to guide future astropharmacy development.

## Introduction

The shift in commercial and government-sponsored spaceflight from Low Earth Orbit (LEO) to long-duration missions lasting multiple years necessitates a fundamental change in the management of pharmaceutical supplies by operators and clinicians. Current pharmaceutical support for LEO spaceflight depends entirely on resupply from Earth, a model that becomes unworkable for exploratory missions that may last three years or more. In LEO missions, medications have already been shown to degrade at higher rates than in terrestrial controls^1,2^. In long-duration missions outside of Earth’s magnetosphere, exposure to Galactic Cosmic Radiation and Solar Particle Events pose additional risks to the molecular integrity of pharmaceuticals through ionization and the generation of free radicals^3^. Based on the current NASA medical formulary which primarily includes *non-peptide* based medications^4^, researchers expect up to 35% of solid oral medications to expire on a three-year mission to Mars^4^. This problem is even more significant for peptide-based (biologic) medications, few of which with relevance to space flight have appreciable shelf lives without refrigeration. Nearly all peptide therapeutics, which typically last one to two years with proper refrigeration, could become unusable^4–6^ for multi-year missions. Pharmaceutical storage and transportation are key limitations of future crewed missions to the Moon and Mars according to NASA Technology Roadmaps - TA 6: Human Health, Life Support, and Habitation Systems, 2015^7^. Peptide medications will require new methods for production and storage if they are to be usable assets for treating spaceflight-related pathologies during multi-year missions^8^.

The duration and environmental constraints of Martian expeditions require greater self-sufficiency for crew members in the development and production of their own pharmaceutical countermeasures to address the expected physiological changes of long-duration spaceflight, including bone density loss, radiation-induced immune dysfunction, and anemia^9–15^. Long-duration LEO missions on the ISS have demonstrated a roughly 1%-1.5% degradation of bone mineral density per month^16^. Teriparatide, a biologic parathyroid (PTH) analog, may be a critical countermeasure to preventing microgravity-induced bone density loss with evidence of efficacy in spaceflight^17^. However, storing it is complex and resource-intensive for a multi-year mission. Similarly, Granulocyte Colony-Stimulating Factor (G-CSF; filgrastim) may be necessary to counteract radiation-induced neutropenia and reduce infection risk for crew members, but faces similar storage and expiration concerns^18^ if transported from Earth at the start of a multi-year mission.

To overcome the resource costs and expiration limitations of transporting compounded pharmaceuticals in orbit, researchers have proposed on-demand production of relevant peptide therapeutics in space. *E. coli* enjoys the most prolific history of terrestrial recombinant peptide therapeutic production^19–23^, and remains a prime vector for therapeutic peptide production in space^8^. *B. subtilis* endospores exhibit remarkable resilience to extreme temperatures, desiccation, radiation, and vacuum environments and represents a prime production platform when coupled with their lengthy research pedigree in microgravity^24–36^. Practical research has demonstrated the feasibility of using *B. subtilis* and *E. coli* to produce peptide therapeutics on-demand^37^. *B. subtilis* is a Gram-positive organism that can secrete proteins, which may further simplify purification. Recent astropharmacy studies confirmed the feasibility of producing biologics, including G-CSF and teriparatide, from *B. subtilis* in laboratory settings^8,37^, demonstrating its potential candidacy for recombinant production for future long-duration space flight.

The novel evaluation matrix proposed in this study guides the objectives of on-demand production of pharmaceutical manufacturing across two domains: operational and clinical relevance (OCR) and recombinant production feasibility (RPF). Operational relevance reflects core spaceflight constraints of power, mass, volume (PMV), and storage limitations, which restrict the practicality of carrying many biologics on multi-year missions. Clinical relevance captures the spectrum of medical importance in spaceflight, prioritizing conditions with a high risk of occurrence and devastating impact on astronaut health and mission completion. In parallel, RPF considers whether a given medication is amenable to in situ production under spaceflight conditions beyond Earth. Collectively, these domains deliver a structured approach for identifying the medications most suitable for on-demand manufacturing during multi-year space missions. The ultimate objective of this matrix is to establish a standardized framework for prioritizing and down-selecting existing pharmaceuticals for on-demand production in space during multi-year missions and to lay a foundation for assessing future peptide medications.

All medications that are primarily composed of amino acids that form peptide chains further organized into one or more proteins will be referred to as “peptide therapeutics” or “peptide medications” for the purposes of this paper. The term “multi-year spaceflight” refers to all missions to the Moon, Mars, or beyond with multi-year architecture which may have few or no resupply opportunities. Implicit in the term “deep space exploration/flight” or “long duration spaceflight” is the supposition that mission duration would be on an order of time that would make resupply of fully formulated medications from Earth very difficult.

## Methods

The original database created by Blum et al. details their methods for the creation of the peptide database for astropharmacy, including their literature review of medications used in previous space missions on the ISS^38^. Several papers published on ISS medications detail the types of medications used since dose tracking was formalized on the ISS^39^, while another provided a more in-depth analysis of expiration dates for medications in the ISS pharmacy^4^. These studies laid the foundation for creating a list of medications analyzed in this study. Further peptide therapeutics were identified from the ThPDB2 database, which provides basic information on every peptide-based medication with known approval by the Federal Drug Administration (FDA) or European Medicine Agency (EMA), including clinical indications and basic protein structure^40^. Medications from any source were excluded if they violated *two* or more of the following criteria:

1. Room temperature (RT) stable for > 36 months
2. Spinal and/or intrathecal route of administration
3. No previous prokaryotic recombinant production
4. No longer FDA/EMA approved without identified clinical trial in the last 10 years
  Medications were also excluded if they violated any *one* of the following criteria:
5. More than one graded criterion is missing across PubChem, FDA, European Medicine Agency (EMA), Electronic Medicine Compendium, Google Scholar, and/or commercial drug labels and data sheets
6. Monoclonal antibodies used for rare metabolic diseases or targeted chemotherapies

However, monoclonal antibodies for common diseases that did not correspond to a targeted chemotherapeutic pathway were considered.

### Exclusion criteria

Room temperature stability for three or more years devalues potential recombinant production in space. Such medications could be more easily taken at launch, assuming non-violation of PMV constraints. Spinal and intrathecal administration remain operationally and clinically risky due to poor control in microgravity and have never been performed in 75 years of spaceflight. No known prokaryotic recombinant production was also a prohibitive criterion because it represents a highly difficult post-translational modification (PTM) that is only known to be performed in eukaryotic cells. Eukaryotic chassis for protein expression are either less likely to withstand the stress of long-duration spaceflight, have not been as thoroughly researched in spaceflight as *E. coli* or *B. subtilis*, or are not commonly used for recombinant therapeutic protein production in commercial or academic settings. Peptide therapeutics that require PTM have been produced in *E. coli*, with additional chemical reactions performed afterwards to achieve functional activity^41–43^. It may ultimately be futile to recombinantly produce therapeutics that would then require one or more PTM that are infeasible to perform in space. However, we still included such therapeutics in our study if there was evidence in the literature of their recombinant production in a prokaryotic chassis (primarily *E. coli*). If the medication is of sufficient clinical relevance to multi-year spaceflight, additional research would still be required to perform PTM with space-suitable-techniques. Several of the biologics identified by Blum et al. were also excluded if they were not currently FDA/EMA approved and had either *never* been FDA/EMA approved or had no history of clinical trials within the last 10 years, as such medications were unlikely to have significant clinical relevance or any foreseeable future approval. Peptide medications that were missing more than one of the predetermined grading criteria after performing the data mining workflow could not be ranked and were also excluded.

### Grading criteria

Grading criteria were derived and divided into two major domains: (1) OCR, and (2) RPF in space. OCR was subdivided into the following criteria for grading:

1. FDA/EMA Approval Status
2. Shelf Life
3. Storage Requirements
4. Risk/Impact Score (RIS) on Human Research Roadmap (HRR)
5. Post-Processing and Purification Requirements

RPF grading criteria were the following:

1. Number of Amino Acids in Peptide Chain
2. Prior Prokaryotic Production
3. Functional Assay
4. Dose Mass Per Day for a 70 kg Patient for Typical Treatment Protocol
5. Presence of Relevant Post-Translational Modification

Every peptide therapeutic meeting inclusion criteria and avoiding exclusion criteria was then subjected to a research workflow with individual searches similar to Blum et al.^38^. The DailyMed website, hosted by the National Library of Medicine, was searched by generic medication name for approval status, shelf-life, storage requirements, clinical indications, and dosing protocols. Relevance to space related pathologies was derived directly from NASA’s HRR risk matrices. Shelf life, dosing protocols, storage requirements, and European approval status were cross-referenced with the EMA website. When multiple brand names and formulations were found for a particular medication, data that granted fewer points on the grading criteria were chosen when related to OCR (i.e., a single formulation of a GLP-1 with a shelf life of three years was chosen, resulting in fewer points, even if several other formulations showed a shelf life of only two years). Data related to RPF that indicated higher scores were always chosen (i.e., a peptide therapeutic produced in *B. subtilis* and *E. coli* was always given two points). This was intended to more accurately reflect currently available options for taking medications in that class directly at launch, while also reflecting a more accurate probability of their successful recombinant production in space. PubChem was searched for structural information and post-translational modifications. PubMed and Google Scholar were searched for evidence of prior production in *B. subtilis*, *E. coli*, and eukaryotic systems. Papers detailing protocols for the recombinant production of the target peptide therapeutic were individually searched for evidence of tagging, and their post-processing and purification protocols were indexed. If a peptide therapeutic had been produced in *B. subtilis* with a His-tag, the search for recombinant production in other chasses was discontinued, as this would grant maximum points for this criterion. Functional assays were searched for in PubMed, Google Scholar, and directly on manufacturers’ websites. If no functional assays were found, a general Google search was performed for any commercial functional assays related to established biochemical pathways using the medications generic name and “functional assay” or “activity assay.” For some therapeutics without regulatory approval, finding papers on Phase I and II trials was difficult and returned no results on Google Scholar or PubMed. When this occurred, OpenEvidence, an AI-based search engine trained specifically on medical literature, was employed as a final option using the same search word combinations employed for Google Scholar or PubMed for obtaining data related to clinical trials. OpenEvidence did not yield any clinical trial results in its database that were not already present on Google Scholar of PubMed, and hence there are no citations in this paper that were retrieved only through use of OpenEvidence.

### Grading scale

This grading matrix was designed as an early-stage formative prioritization matrix rather than a reflective unidimensional scale. The criteria were selected a priori to represent distinct operational, clinical, and manufacturing constraints relevant to multi-year spaceflight. Equal weighting was used deliberately as a neutral starting assumption because there is currently no empirical basis or expert-consensus model to justify differential weights across these heterogeneous criteria, and unequal weight could introduce spurious precision. We further balanced OCR and RPF domains because both mission relevance and in situ manufacturability are necessary conditions for prioritizing candidates for space-based recombinant production. To examine whether this assumption disproportionately influenced results, we performed a leave-one-criterion out sensitivity analysis to detect if highest-ranked candidates remained stable when each individual criterion was removed from consideration.

### FDA approval and clinical trial status

Regulatory (FDA/EMA) approval provides clinicians with a snapshot of the medication’s current regulatory status, which indicates its clinical relevance to a specific pathology and history of clinical testing. Numerous medications are used “off-label” for pathologies that were not enumerated in the initial approval process. These medications are still approved for use in some pathologies, and their pharmacodynamics and kinetics are often well understood, at least under the conditions for which the medications were initially approved. Peptide medications which are particularly novel may not have had sufficient time for study to be fully approved at the time of writing and hence were included if they had also had a clinical trial (Phase I or greater) in the last decade. If a medication was fully and currently approved for any use, it was given a score of two points. If it was not approved but had a clinical trial in the last ten years, it received a score of one. Medications that were not approved and had no clinical trial in the last ten years, or had lost approval, were given zero points.

### Shelf life

A driving factor for directing research resources towards on-demand astropharmacy during multi-year space missions is the difficulty of taking medications from Earth. While a trans-Martian flight trajectory timeframe is well understood in current operational planning^13,44^, it is not known how much PMV will be allocated to medication storage. It may not be possible to take many medications to deep space at all. However, it stands to reason that medications associated with high-risk/high-impact pathology *and* that are easily taken from Earth because of their long shelf stability and low PMV requirements would be the most likely to be taken from the start of the mission to be used when needed. Hence, medications with particularly *short* shelf life are relatively good candidates for on-demand production, as taking them from launch is a waste of PMV. Peptide medications with shelf lives of less than 12 months were given two points, and medications with shelf lives of 12-36 months were given one point. Medications with shelf lives exceeding 36 months were given zero points.

### Storage requirements

Closely related and of similar importance to shelf life are storage requirements, particularly refrigeration requirements. Medications requiring significant refrigeration necessitate a large increase in secondary PMV requirements for a refrigeration unit. This problem is further compounded for medications that require freezing. Medications that also require refrigeration are candidates for on-demand production, as it is unlikely that PMV budgets would permit storage at 2 °C to 8 °C for multiple years, or frozen at −20 °C to −80 °C which is typical of peptide therapeutics (see Supplementary Data 01, and Supplementary Table 1 for citations). Medications that require storage below 0 °C were given two points. Because refrigeration, rather than freezing, requires less PMV, medications that are stored at 2 °C to 8 °C were given one point. Medications that could be stored at room temperature were given no points.

### Risk/impact score on human research roadmap

Since PMV are likely to be severely limited in deep space travel, medications that prevent or treat the unique consequences of multi-year space flight should be prioritized when building the astropharmacy. The HRR currently details risks, gaps, and tasks related to major space-related pathologies and medical care. Because the HRR was developed by a committee of experienced flight surgeons and physicians, we considered it to be the best available analysis of the risk and impact score (RIS) of the pathologic consequences of spaceflight. Conveniently, the risk and impact components are combined by HRR into a single score in the HRR matrices and regraded after current mitigation efforts are applied. Post-mitigated RIS is color-coded as either green, yellow, or red, corresponding to low, medium, and high RIS, respectively. Space-related pathologies for which RIS was red *for long-duration* spaceflight require significant advancement before a tolerable post-mitigation RIS is achieved. Medications linked to red RIS were given two points, and those linked to yellow RIS were given one point. Medications associated with low RIS were given zero points.

### Protein post-processing and purification

Post-processing and purification are essential for achieving the medication purity suitable for clinical use. Previous terrestrial astropharmacy efforts have relied on techniques such as sonication, centrifugation, and quantification assays^8,37^, which require benchtop hardware that may not be feasible for multi-year space missions due to PMV constraints. Some approaches to astropharmacy peptide production methods have also depended on reagents with shorter shelf lives than the medications themselves^8,37^. The ability to produce a protein in space offers little advantage if the required purification methods are unsuitable for long-duration missions. Peptide therapeutics with a history of being processed using shelf stable techniques (>3years) are therefore more feasible for space-based production.

Histidine (His) tags remain one of the most convenient and widely used purification strategies in academic settings and industry^45–49^. His-tagged proteins can be captured using nickel-nitrilotriacetic acid (Ni-NTA) resins via immobilized metal affinity chromatography (IMAC), which uses compact, shelf-stable columns^45,48,50^. This approach has been demonstrated in suitcase-sized pharmaceutical production systems and in prior *B. subtilis* astropharmacy studies^37,51^. Although few FDA-approved drugs retain His-tags due to immunogenicity concerns, several His-tagged monoclonal antibodies are approved^52^, while evidence on His-tag immunogenicity remains somewhat mixed and limited to mice^53–55^.

Direct secretion of proteins to the extracellular space may further reduce hardware requirements by avoiding mechanical cell lysis. The use of self-cleaving tag systems^45,46,56–58^ may also eliminate additional purification steps. However, some purification will always be required to achieve pharmaceutical-grade products. Alternative affinity tags, such as cellulose-binding domains, also use shelf-stable resins^59^, but are less common in pharmaceutical manufacturing^45^ and lack astropharmacy-specific precedent. Accordingly, therapeutics with a history of His-tag purification were given two points, those using sugar-binding domains were given one point, and those requiring short-lived reagents were given zero points.

### Amino acid chain length

Amino acid (AA) chain length and overall mass of a peptide chain, often measured in kDa, relate closely to the ability of a prokaryotic system to produce and secrete the peptide therapeutic^60,61^. Different protein expression systems have differing considerations for protein secretion. In *B. subtilis*, proteins that are too small risk deconstruction by cytoplasmic proteolysis, whereas peptide therapeutics that are over 200 AA may have difficulty being secreted at all or in a properly folded conformation^62,63^. Proteins that can be secreted in a folded form may be limited to 100 AA or fewer, as they cannot typically fit through the secretion channel in the host cell membrane^64–66^. Recombinant production with lysis and collection is an option for some, if not all, space produced peptide therapeutics, but would most likely require more equipment and/or purification steps. Without knowing the optimal production and purification methods for each peptide therapeutic specifically for space, we hypothesize that smaller proteins will tend to have more options for recombinant production and purification in space. Peptide therapeutics < 50 AA were given two points, those between 50 and 200 AA were given one point, and peptide therapeutics > 200 AA were given zero points.

### Prior transgenic production

Numerous methods are used to produce therapeutic peptides. In particular, very small peptide chains tend to be produced by solid-phase peptide synthesis (SPPS)^67–70^, whereas others are produced exclusively in eukaryotic expression systems, which can facilitate complex post-translational modifications^71^. While there are some yeasts and slime molds that have been studied that can withstand the extreme environment of space^35,72,73^, they generally do not grow as quickly, produce as much protein, or have as rich space flight or academic heritage as *E. coli* or *B. subtilis*. *E. coli* can also produce some peptide therapeutics, which then require additional chemical reactions to achieve the PTM required for therapeutic functionality^43,74^. It is theoretically possible that cell-based protein expression systems other than *B. subtilis* will be used for on-demand astropharmacy. However, *B. subtilis* remains one of the most studied protein-producing bacteria in space and is a major protein producer in both therapeutic and industrial industries^75^. Given its ability to sporulate, protect its genome from radiation, and withstand extremes of temperature and pressure^24–36^, *B. subtilis* is most likely a superior candidate to *E. coli*, which does not sporulate and is less resilient to the extremes of spaceflight. Because *E. coli* is the most studied recombinant protein producer in the pharmaceutical industry overall, it still remains a superb model for future recombinant therapeutic peptide production in space, even if it is not the optimal chassis for multi-year missions. Peptide therapeutics with a history of recombinant production in *B. subtilis* were given two points as this indicates the current existence of production protocols. Peptide therapeutics with a recombinant production history in another prokaryotic chassis were given one point, as it is more likely that these peptides can be produced in *B. subtilis*. Peptide therapeutics with an exclusive production history in eukaryotic expression systems or with SPPS were given zero points.

### Functional assays

Functional (activity) assays play a vital role in ensuring that peptide therapeutics perform as intended. Creating protein therapeutics in space will be a complex task with many variables affecting the finished product. Quality control methods are required to ensure that the product is appropriate for patient administration. Peptide therapeutics with functional assays that do not require cultured cell lines (which are most likely not feasible for multi-year spaceflight) for activity reporting were thus given two points. Peptide therapeutics identified as using cell-based functional assays were given one point. Peptide therapeutics with an exclusive history of activity assays in animal models are particularly undesirable because of PMV and crew time considerations and were given zero points. Some peptide therapeutics, such as fibrinolytics, lie within a gray area. While these peptide therapeutics have cell- and non-cell-based functional assays, it is also possible to simply measure their activity using a blood sample taken from the patient. In these situations, peptide therapeutics were given two points.

### Production of target dosing

Different peptide therapeutics require different production outputs to meet “dose-in-a-day” targets. For instance, target production of a vasopressor medication in a given time will be on the order of grams^76^, which will likely be much more difficult than producing a small <1 mg dose of therapeutics a day for G-CSF^77^. Producing the requisite amount of a specific therapeutic within an appropriate timeframe may be impractical for certain medications. However, it is very difficult to know which will turn out to be truly infeasible versus being extremely difficult prior to optimizing protocols terrestrially and in space. We used production data from several papers^8,37,51^ as benchmarks for what may be feasible to produce by a *B. subtilis* expression system, with the understanding that *B. subtilis* or any protein expression system used in space could likely reach target output production if extensively optimized for each medication. With that in mind, therapeutics that require < 100 mcg/day for dosing in a 70 kg patient will typically require fewer cellular production inputs and post-processing resources to achieve, whereas therapeutics that require large doses to reach therapeutic effect will be relatively more difficult to produce. Therapeutics that require <100 mcg/day dose for a 70 kg patient for associated space-related pathology were given two points. Therapeutics requiring between 100 mcg/day and 500 mcg/day were given one point, and those requiring > 500 mcg/day were given no points.

### Post-translational modifications (PTM)

Many proteins require complex modifications after their expression in the cell to achieve full biochemical functionality at their target receptors in the patient. Common PTMs in human peptide therapeutics include multi-domain assembly, glycosylation, disulfide bonding between cysteine residues, or amidation^71^. Many peptide therapeutics are recombinantly produced in Chinese hamster ovary (CHO) cells or with a eukaryotic protein expression system because prokaryotic systems lack the cellular machinery to make these PTMs. Some therapeutics, such as G-CSF, are glycosylated in their endogenous forms but function well without glycosylation, which is subsequently omitted in recombinant production^78^. Although eukaryotic chasses, such as *P. pistoris* and *C. cerevisiae* do have some spaceflight testing history^35,72,73,79–81^, they do not possess as comprehensive academic heritage in space compared to *B. subtilis*, which has benefited from panspermia research particularly when it comes to assessing resilience to the space environment^24–36^. Some peptide therapeutics can be recombinantly produced in prokaryotes and have PTMs added in vitro to make them functional^43,74^. While any in vitro PTM produced by a prokaryotic chassis would be difficult in space, it is theoretically possible in some instances. Peptide therapeutics with no PTMs or functionally irrelevant PTMs were given two points. Peptide therapeutics with one PTM were given one point, and those with several PTMs were given zero points.

### Statistical analysis

OCR and RPF criteria were selected a priori to capture distinct OCR and RPF constraints. Given the ordinal scoring rubric, pairwise Spearman correlation matrices were generated in RStudio for the OCR and RPF domains. Correlation scores for this metric range from −1 to +1, indicating perfect negative and positive correlation, respectively, to other criteria, and scores near zero representing less correlation. A leave-one-out sensitivity analysis was then conducted for each domain by removing each OCR and RPF criterion one at a time to measure the effect of each individual criterion on the overall rankings of the medications.

## Results

### Medications meeting inclusion criteria

**Fig. 1:**
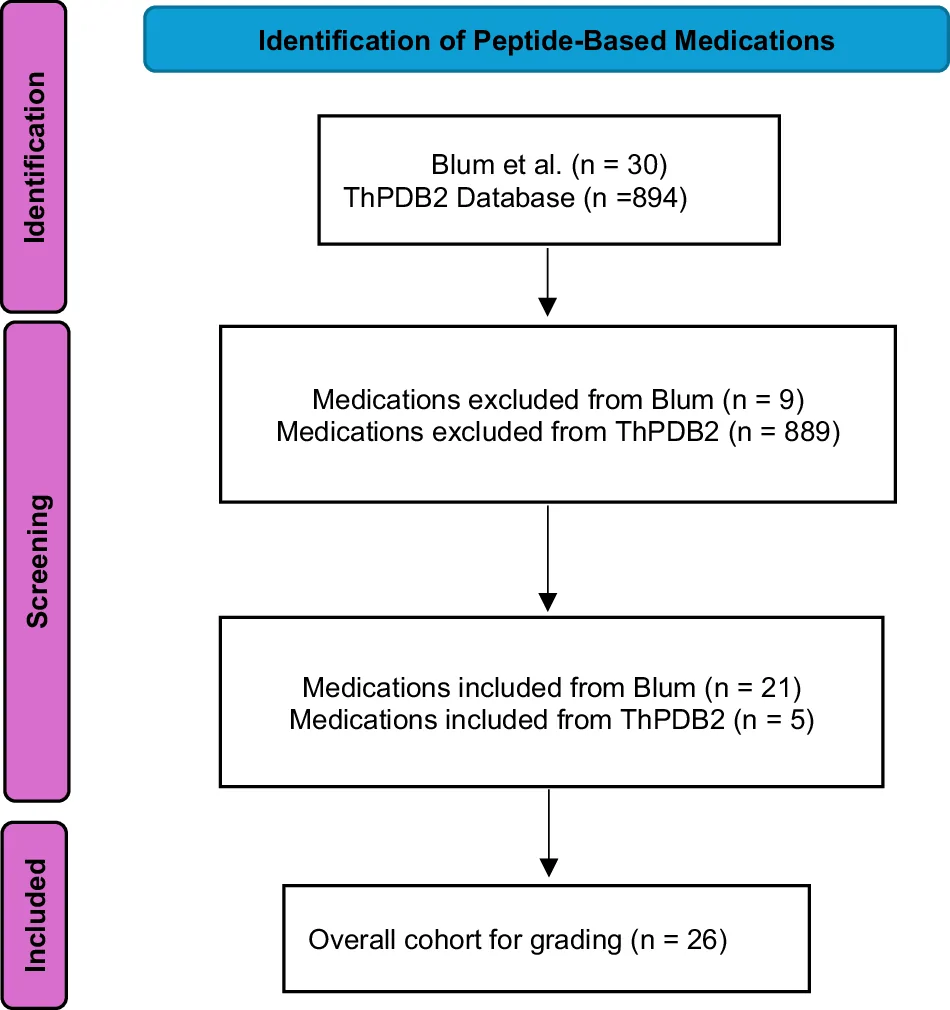
Diagram of included and excluded medications. Medications excluded from Blum et al. (*n* = 9) included Factor VII, teicoplanin, ziconotide, CBLB502, CBLB612, PL-18, nesiritide, leptucin, and PL-18. Medications excluded from ThPDB2 (*n* = 882) consisted primarily of repeat entries of the same drug within the database, monoclonal antibodies targeting rare metabolic conditions or chemotherapeutic pathways, and medications already identified by Blum et al. Medications included from ThPDB2 (*n* = 5) were Factor VIII, Factor IIa, eptifibatide, and bivalirudin. We also included denosumab, a monoclonal antibody, due to its relevance to space flight-related bone atrophy and its usage in osteoporosis, which is a *common* vice *rare* condition.

Results of included medications are in Fig. 1.

Results of medications that were excluded from the Blum database along with the exclusion criteria they violated are seen in Table 1.

**Table 1:** Medications excluded after application of exclusion criteria

Medication	≥36 months RT Stable	Spinal / Intrathecal ROA	No prokaryotic production	No FDA/EMA approval or recent trial	>1 graded criterion missing
Factor VII	✓		✓		
Teicoplanin	✓	✓	✓		
Ziconotide		✓	✓		
Nesiritide	✓			✓	
Hirudin				✓	
Leptucin			✓	✓	
CBLB502					✓
CBLB612			✓		✓
PL-18			✓		✓

Medications from the Blum database that were excluded.

*RT* room temperatue, *ROA* route of administration.

### OCR and RPF winners and losers

From the perspective of operational and clinical relevance (OCR), teriparatide, abaloparatide, amylin, and angiotensin II each scored the highest with 8/10 points. These medications are approved by FDA/EMA, have clinically relevant connections to the HRR risk matrices, require refrigeration, and have relatively poor shelf stability. They also had a history of purification methods that are more likely to be stable during multi-year missions. The lowest performing medication in the OCR domain was bivalirudin at 5/10, which has multi-year shelf stability at room temperature and is only produced via SPPS, making it relatively unsuitable as a candidate for expression in space.

From the perspective of recombinant production feasibility (RPF), G-CSF and amylin scored second highest at 8/10, while teriparatide scored the highest at 9/10. Both medications have small peptide chains that can be expressed by either direct secretion into the cytoplasm or via cell lysis and collection. They also have a history of expression in *B. subtilis*, a promising expression system for multi-year missions because of its extensive scientific pedigree in space and resilience to the space environment^24–36^. Lastly, G-CSF and parathyroid analogs are also dosed on small enough scales to make production of therapeutic doses more achievable^82–84^, which also contributed to their high RPF scores. When considering both OCR and RPF together, teriparatide was the overall winner, scoring 17/20, with abaloparatide and amylin close seconds with scores of 16/20. Angiotensin II, daptomycin, GLP-1 agonists, G-CSF, GM-CSF, and salmon calcitonin tied for third place scoring 14/20. The results are seen in Table 2.

**Table 2:** OCR and RPF scores of peptide medications

Pharmaceutical Name	Mechanism	Drug class	Spaceflight Relevance	OCR	RPF	Total Score
Teriparatide	Stimulates bone formation	*Parathyroid hormone analog*	Bone loss	8	9	**17**
Abaloparatide	Stimulates osteoblast activity	*Parathyroid hormone analog*	Bone loss	8	8	**16**
Amylin (Pramlintide)	Amylin and calcitonin agonist	*Osteoporosis medications*	Counteract bone loss	8	8	**16**
Daptomycin	Inhibits bacterial cell walls	*Antibiotic*	In-flight infections	7	7	**14**
Granulocyte-Macrophage Colony-Stimulating Factor (GM-CSF)	Stimulates hematopoietic progenitor cells	*Colony growth stimulating factor*	Radiation-induced lung injury	7	7	**14**
GLP-1 agonists semaglutide	Decreases cardiac remodeling	*GLP-1 agonist*	Cardiac remodeling prevention	7	7	**14**
Salmon Calcitonin	Calcitonin receptor agonist	*Osteoporosis medications*	Counteract bone loss	7	7	**14**
Angiotensin II	Constricts renal arterioles	*Vasopressor*	Hypotension	8	6	**14**
Granulocyte -Colony Stimulating Factor (G-CSF)	Stimulates granulocytes in bone marrow	*Colony growth stimulating factor*	Radiation-induced neutropenia	6	8	**14**
Insulin	Facilitates intracellular transport of glucose and potassium	*Metabolic hormone*	None specified	7	6	**13**
Streptokinase	Plasminogen activator	*Fibrinolytic*	Dissolution of blood clots	7	6	**13**
Rusalatide Acetate (TP508)	Activates radioresistant stem cells in GI tract	*Regenerative peptide*	Radiation-induced GI damage	6	7	**13**
Glucagon	Stimulates glycogenolysis and gluconeogenesis	*Metabolic hormone*	None specified	6	6	**12**
Factor IIa (Topical Thrombin)	Coagulation cascade factor	*Coagulant*	Local hemorrhage control	8	4	**12**
tPA	Plasminogen activator	*Fibrinolytic*	Dissolution of blood clots	7	5	**12**
IL-18 Binding Protein	Inhibits IL-18 mitigating radiation damage	*Cytokine*	Radioprotectant	8	3	**11**
ADH	Vasoconstriction and water resorption	*Vasopressor*	Hypotension	6	5	**11**
IL-11	Stimulates production of platelets	*Thrombopoietic growth factor*	GI radioprotectant	5	5	**10**
Hirudin	Direct thrombin inhibitor	*Anticoagulant*	Venous thrombo-embolism (VTE)	5	5	**10**
Erythropoietin	Stimulates production of erythrocytes	*Hematopoietic growth factor*	Acute radiation syndrome	5	5	**10**
Delta Sleep Inducing Peptide	Improves sleep latency	*Sleep aid*	Insomnia	5	5	**10**
Romiplostim	Increases platelet and megakaryocyte production	*Thrombopoietin receptor agonist*	Radiation-induced thrombocytopenia	6	4	**10**
Bivalirudin	Direct thrombin inhibitor	*Anticoagulant*	VTE	5	4	**9**
Eptifibatide	GPIIb/IIIa inhibitor	*Antiplatelet*	VTE	6	2	**8**
Factor VIII	Coagulation cascade factor	*Procoagulant*	Systemic hemorrhage control	6	2	**8**
Denosumab	Antibody against RANKL	*Osteoporosis medications*	Counteract bone loss	5	2	**7**

Maximum domain score = 10 for each domain, with a total possible score of 20 points. Citations for each medication are included in Supplementary Table 1. Medications are organized by total score. For medications with equaling scores are organized then by OCR score alphabetically, followed by RPF scores alphabetically.

*OCR* operational and clinical relevance, *RPF* recombinant production feasibility.

### OCR and RPF general trends

Operational and clinical relevance scores clustered within a narrow range of 5–8 points. This was expected as it reflects the effects of the exclusion criteria of (1) FDA/governing agency approval of the medication list *and* (2) Shelf stability of greater than 36 months at room temperature. Recombinant production feasibility was a much more heterogeneous domain, with scores ranging from 2–9 points.

Pairwise Spearman correlations within the OCR domain ranged from rho (ρ) values of −0.56 to 0.36, while correlations within the RPF domain ranged from −0.53 to 0.43. The strongest observed associations were between FDA approval and shelf life in the OCR domain (ρ = −0.56), and between functional assay and dose requirements in the RPF domain (ρ = −0.53). Full Spearman correlation matrices can be seen for OCR (Table 3) and RPF (Table 4). Leave-one-out sensitivity analysis for OCR (Table 5) and RPF (Table 6) showed that the identity of the top-ranked candidates remained stable when individual RPF criteria were removed, indicating that rankings were not driven by any single criteria. Complete rankings for the sensitivity analysis are provided within Supplementary Data 01.

**Table 3:** Spearman correlation matrix for OCR grading criteria

Criterion	FDA Approval	Shelf Life	Storage Requirements	HRR Risk/Impact	Post-Processing & Purification
FDA Approval	1.00	−0.56	−0.36	0.36	0.09
Shelf Life	−0.56	1.00	0.32	−0.25	−0.08
Storage Requirements	−0.36	0.32	1.00	−0.40	−0.40
HRR Risk/Impact	0.36	−0.25	−0.40	1.00	−0.06
Post−Processing & Purification	0.09	−0.08	−0.40	−0.06	1.00

Spearman’s rank correlation coefficient ranges from −1 to +1, where +1 indicates a positive correlation, and −1 a negative correlation. Values near 0 indicate no correlation.

**Table 4:** Spearman correlation matrix for RPF grading criteria

Criterion	Amino Acids	Prior Transgenic Production	Functional Assay	Dose for 70 kg	PTM
Amino Acids	1.00	0.15	−0.29	0.31	0.43
Prior Transgenic Production	0.15	1.00	−0.11	0.04	0.32
Functional Assay	−0.29	−0.11	1.00	−0.53	−0.20
Dose for 70 kg	0.31	0.04	−0.53	1.00	0.03
PTM	0.43	0.32	−0.20	0.03	1.00

Spearman’s rank correlation coefficient ranges from −1 to +1, where +1 indicates a positive correlation, and −1 a negative correlation. Values near 0 indicate no correlation.

**Table 5:** Medication scores with individual OCR grading criteria removed

Rank	Without Amino Acid Count	Without Prior Transgenic Production	Without Functional Assay	Without Dosing	Without Post Translational Modifications
1^st^	Teriparatide	Teriparatide	Teriparatide	Teriparatide	Teriparatide
2^nd^	Abaloparatide	Abaloparatide	Abaloparatide	Abaloparatide	Abaloparatide
3^rd^	Amylin	Amylin	Amylin	Amylin	Amylin
4^th^	Angiotensin II	G-CSF	GLP-1	Daptomycin	TP508

Sensitivity analysis showing changes in medication rankings when individual OCR criteria are removed.

**Table 6:** Medication scores with individual RPF grading criteria removed

Rank	Without Regulatory Approval	Without Prior Shelf Life	Without Storage Requirements	Without HRR Risk/Impact Score	Without Post Processing
1^st^	Amylin	Amylin	Amylin	Teriparatide	Amylin
2^nd^	Teriparatide	Abaloparatide	Teriparatide	Abaloparatide	Teriparatide
3^rd^	Abaloparatide	Teriparatide	Abaloparatide	Amylin	Abaloparatide
4^th^	Angiotensin II	Angiotensin II	Angiotensin II	Angiotensin II	GLP-1

Sensitivity analysis showing changes in medication rankings when individual RPF criteria are removed.

## Discussion

This is the first study to create a prioritization framework for on-demand recombinant production of peptide therapeutics in space during multi-year space missions based on operational and clinical relevance and recombinant production feasibility. Previous studies aiming at on-demand production of peptide therapeutics for astropharmacy were not based on a particular methodology for selection. Our study expanded the database of space-relevant peptide therapeutics as well, while providing a method to rank future peptide therapeutics.

In both domains, most pairwise associations were weak to moderate in magnitude, with no correlation approaching unity, indicating limited redundancy among the included criteria. These correlation patterns support the interpretation that the selected criteria capture related but non-redundant dimensions within each domain and are consistent with the intended use of the framework as a prioritization tool with distinct operational and manufacturing considerations. This was further supported by candidate therapeutic ranking stability across OCR and RPF domains even when individual grading criteria were removed. The persistence of candidate rankings across leave-one-out sensitivity analyses supports the use of OCR and RPF as distinct but complementary domains, indicating that prioritization reflects an aggregate of operational and manufacturing considerations rather than dependence on any single grading criterion.

Future efforts for astropharmacy should be aimed at expanding the cell-based capabilities of *B. subtilis* because of its proven flight history and success as a protein producer in industry^75,85^. Particularly, strains such as WB800N are particularly promising for producing and secreting medications because of their extensive extracellular protease knockouts^86^. Strategies should avoid processing or purification that are infeasible in multi-year spaceflight. Some methods may include making use of His-tags with methods of self-cleavage or cleavage via shelf-stable reagents such as SNAC cleavage^48,49,56,87^. If direct secretion is not compatible with a particular peptide therapeutic, an inducible autolytic system may also have to be developed to better facilitate lysate harvesting. Protein folding requirements will also prove challenging for future recombinant therapeutic production in space. Eukaryotic chasses with some proven resilience to the space environment, such as *P. pistoris* and *C. cerevisiae*^79–81^, may prove valuable protein producers capable of performing PTM required of some space-relevant therapeutics^88^.

This study has several limitations. Based on our grading criteria, the priority peptides include therapeutic candidates with parathyroid analogs, amylin, calcitonin, GLP-1 agonists, G-CSF, and GM-CSF. Our study considered similar medications in the same class when available and obvious on DailyMed, EMC, PubChem, and the peptide databases of Blum et al. and ThPDB2. However, an exhaustive search of brand-name formulations of the same medication was not performed. Although we could expect similar dosing quantities and therapeutic effects for different brands of the same medication, we might expect different excipients or packaging, which may impact shelf life and storage requirements in microgravity. A general limitation of this framework is that equal weighting was used as a pragmatic baseline in the absence of empirical or consensus-derived weights, and future work should refine criterion weights using expert elicitation, pairwise comparison, or other formal multi-criteria decision analysis methods.

There are many other OCR grading criteria that were not included because they are unknown at this time and relatively difficult to estimate. Such criteria that would certainly impact OCR and would benefit from future cost-benefit analyses include PMV restrictions, crew time and experience required for peptide production, emerging purification methods^46,56^, and employment of alternative countermeasures for space-related pathologies that obviate the need for medications. Development of novel storage methods that extend the shelf life of medications taken from launch may also drastically change the strategy for providing on-demand astropharmacy. Many medications are intact and usable long after the manufacturer’s stated shelf life^89^, but this study did not attempt to consider available data or how this might impact astropharmacy strategy.

Limitations of our RPF criteria include the consideration of eukaryotic chassis with high resilience to the space environment that are capable of complex post-translational modifications required for relevant peptide therapeutics^35,72,73^. While eukaryotic chasses are limited for the reasons explained in the “Prior Transgenic Production” section of this study, we did not make a concerted effort to consider the potential of this production strategy due to the paucity of flight heritage compared to *E. coli* and *B. Subtilis*. Additional research is warranted to assess the feasibility of eukaryotic chasses that have space flight heritage, particularly *P. pistoris*, and *C. cerevisiae* as protein producers capable of performing PTMs. Additionally, mRNA-based therapeutic technologies were not included in this analysis because of their scarce clinical use outside vaccine technology^90^. However, mRNA therapies seem particularly well-suited for spaceflight as they could potentially require only a single or periodic dosing, which would decrease the amount of peptide production required to achieve therapeutic effects. Owing to their inherent instability, mRNA therapeutics used years into a long-duration mission would most likely require on-demand production. This paper does not examine other key quality-control requirements for protein therapeutics, such as purity and aggregation, process-related impurities, and stability post-production. Furthermore, this study does not address excipient selection, or compatibility with available medication delivery systems in space as matters of medication formulation. The authors recognize that these are critical aspects of therapeutic production and delivery in space, and recommend them as topics for future research, and expansion of the grading framework. Lastly, although monoclonal antibodies were largely excluded from this study, Fab and Fc antibody fragments can be produced in prokaryotic systems with therapeutic effects^91–93^. We did not attempt to include these in this study. While several FDA-approved Fc or Fab fragments are produced by prokaryotes^94^, abciximab is the only one with space flight relevance that was obvious without a systemic review and is only produced in eukaryotic cells^95^.

## Supplementary information

Supplementary Table 1

Supplementary Data 01

## Data Availability

All data generated or analyzed during this study are included in this published article and supplementary material.
