# Supplementary material for: On-demand peptide therapeutics for multi-year space exploration: analysis of clinical and operational relevance and recombinant production feasibility
Source: NPJ Microgravity. 2026 Jul 9;12:92. doi: 10.1038/s41526-026-00630-z (PMC13724685; doi:10.1038/s41526-026-00630-z)
Supplement: Supplementary file 1 [file 41526_2026_630_MOESM1_ESM.pdf]

Supplementary Table 1| Supporting References for Supplementary Data 01

Pharmaceutical Name	References
Teriparatide	[1–10]
Abaloparatide	[11–22]
Amylin (pramlintide)	[23–27,27–30,30–39]
Angiotensin II	[40–49]
Daptomycin	[50–59]
GLP-1 agonists (semaglutide)	[60–70]
G-CSF	[1,71–78]
GM-CSF	[79–87]
Salmon Calcitonin	[88–97]
Insulin	[98–111]
Rusalatide Acetate (TP508)	[50,112–123]
Streptokinase	[124–132]
Factor IIa (topical thrombin)	[133–140]
Glucagon	[66,67,141–151]
tPA	[152–160]
ADH	[48,161–166]
IL-18 Binding Protein	[167–179]
Delta Sleep Inducing Peptide	[180–188]
Erythropoietin	[189–200]
Hirudin	[201–205]
IL-11	[206–221]
Romiplostim	[222–227]
Bivalirudin	[228–232]
Eptifibatide	[233–236]
Factor VIII	[237–242]
Denosumab	[243–247]

These are supporting references corresponding to the medications in Supplementary Data 01 across OCR and RPF grading criteria.

References for Supplementary Table 1

1. Vallota-Eastman, A. *et al.* Bacillus subtilis engineered for aerospace medicine: a platform for on-demand production of pharmaceutical peptides. *Front. Space Technol.* **4**, (2023).
2. DailyMed - TERIPARATIDE injection, solution. https://dailymed.nlm.nih.gov/dailymed/drugInfo.cfm?setid=1b007339-dd0d-f019-5e0a-9b1b0f75011c.
3. Osteoporosis Injection Treatment | FORTEO® (teriparatide injection). https://forteo.lilly.com/.
4. Walker, M. D. & Shane, E. Postmenopausal Osteoporosis. *N. Engl. J. Med.* **389**, 1979–1991 (2023).
5. Qaseem, A., Hicks, L. A., Etxeandia-Ikobaltzeta, I., Shamliyan, T. & Cooney, T. G. Pharmacologic Treatment of Primary Osteoporosis or Low Bone Mass to Prevent Fractures in Adults: A Living Clinical Guideline From the American College of Physicians. *Ann. Intern. Med.* **176**, 224–238 (2023).
6. Lindsay, R., Krege, J. H., Marin, F., Jin, L. & Stepan, J. J. Teriparatide for osteoporosis: importance of the full course. *Osteoporos. Int. J. Establ. Result Coop. Eur. Found. Osteoporos. Natl. Osteoporos. Found. USA* **27**, 2395–2410 (2016).
7. Hamedifar, H. *et al.* A Novel Approach for High Level Expression of Soluble Recombinant Human Parathyroid Hormone (rhPTH 1-34) in Escherichia coli. *Avicenna J. Med. Biotechnol.* **5**, 193–201 (2013).
8. Bakhtiari, N., Amini Bayat, Z., Sagharidouz, S. & Vaez, M. Overexpression of Recombinant Human Teriparatide, rhPTH (1-34) in Escherichia coli : An Innovative Gene Fusion Approach. *Avicenna J. Med. Biotechnol.* **9**, 19–22 (2017).

9. Xu, D., Wu, H. & Zhou, C. Fusion of parathyroid hormone (1–34) to an albumin-binding domain improves osteogenesis. *J. Drug Deliv. Sci. Technol.* **79**, 104019 (2023).

10. Flórez, V., Marizcurrena, J. & Laviña, M. Secretion of the human parathyroid hormone through a microcin type I secretion system in Escherichia coli. *Microb. Cell Factories* **23**, 273 (2024).

11. Wang, H. *et al.* Recombinant Human Parathyroid Hormone Related Protein 1-34 and 1-84 and Their Roles in Osteoporosis Treatment. *PLOS ONE* **9**, e88237 (2014).

12. Czerwinski, E. *et al.* The Efficacy and Safety of Abaloparatide-SC in Men With Osteoporosis: A Randomized Clinical Trial. *J. Bone Miner. Res. Off. J. Am. Soc. Bone Miner. Res.* **37**, 2435–2442 (2022).

13. Xu, F., Wang, Y. & Zhu, X. The Safety and Efficacy of Abaloparatide on Postmenopausal Osteoporosis: A Systematic Review and Meta-analysis. *Clin. Ther.* **46**, 267–274 (2024).

14. Makino, A. *et al.* Frequent administration of abaloparatide shows greater gains in bone anabolic window and bone mineral density in mice: A comparison with teriparatide. *Bone* **142**, 115651 (2021).

15. Hong, P. *et al.* Is abaloparatide more efficacious on increasing bone mineral density than teriparatide for women with postmenopausal osteoporosis? An updated meta-analysis. *J. Orthop. Surg.* **18**, 116 (2023).

16. Beaudart, C. *et al.* PTH1 receptor agonists for fracture risk: a systematic review and network meta-analysis. *Osteoporos. Int.* **36**, 951–967 (2025).

17. DailyMed - TYMLOS- abaloparatide injection, solution. https://dailymed.nlm.nih.gov/dailymed/drugInfo.cfm?setid=712143d9-e21e-4013-bb3b-3426a21060a8.

18. Eladynos | European Medicines Agency (EMA). https://www.ema.europa.eu/en/medicines/human/EPAR/eladynos (2022).

19. Le Henaff, C. *et al.* Abaloparatide at the same dose has the same effects on bone as PTH (1-34) in mice. *J. Bone Miner. Res. Off. J. Am. Soc. Bone Miner. Res.* **35**, 714–724 (2020).

20. Hammonds, R. G. *et al.* Purification and characterization of recombinant human parathyroid hormone-related protein. *J. Biol. Chem.* **264**, 14806–14811 (1989).

21. Liu, Q. *et al.* Large scale preparation of recombinant human parathyroid hormone 1–84 from Escherichia coli. *Protein Expr. Purif.* **54**, 212–219 (2007).

22. Rabbani, S. A. *et al.* Expression and characterization of recombinant rat parathyroid hormone-related peptide (1–141) and an amino-terminally-truncated analogue (38–141). *Mol. Cell. Endocrinol.* **130**, 13–21 (1997).

23. Ryan, G. J., Jobe, L. J. & Martin, R. Pramlintide in the treatment of type 1 and type 2 diabetes mellitus. *Clin. Ther.* **27**, 1500–1512 (2005).

24. Knop, F. K. *The Role of Amylin in Bone Metabolism*. https://clinicaltrials.gov/study/NCT06186063 (2024).

25. PubChem. Islet Amyloid Polypeptide. https://pubchem.ncbi.nlm.nih.gov/compound/16132430.

26. PubChem. IAPP - islet amyloid polypeptide (human). https://pubchem.ncbi.nlm.nih.gov/gene/IAPP/human.

27. DailyMed - SYMLINPEN- pramlintide acetate injection. https://dailymed.nlm.nih.gov/dailymed/drugInfo.cfm?setid=4aea30ff-eb0d-45c1-b114-3127966328ff.

28. Frandsen, C. S., Dejgaard, T. F. & Madsbad, S. Non-insulin drugs to treat hyperglycaemia in type 1 diabetes mellitus. *Lancet Diabetes Endocrinol.* **4**, 766–780 (2016).

29. Holt, R. I. G. *et al.* The Management of Type 1 Diabetes in Adults. A Consensus Report by the American Diabetes Association (ADA) and the European Association for the Study of Diabetes (EASD). *Diabetes Care* **44**, 2589–2625 (2021).

30. Bower, R. L. & Hay, D. L. Amylin structure-function relationships and receptor pharmacology: implications for amylin mimetic drug development. *Br. J. Pharmacol.* **173**, 1883–1898 (2016).

31. Krampert, M. *et al.* Amyloidogenicity of recombinant human pro-islet amyloid polypeptide (ProIAPP). *Chem. Biol.* **7**, 855–871 (2000).

32. Noh, D., Bower, R. L., Hay, D. L., Zhyvoloup, A. & Raleigh, D. P. Analysis of Amylin Consensus Sequences Suggests That Human Amylin Is Not Optimized to Minimize Amyloid Formation and Provides Clues to Factors That Modulate Amyloidogenicity. *ACS Chem. Biol.* **15**, 1408–1416 (2020).

33. Borm, A. K. *et al.* The Effect of Pramlintide (Amylin Analogue) Treatment on Bone Metabolism and Bone Density in Patients with Type 1 Diabetes Mellitus. *Horm. Metab. Res.* **31**, 472–475 (1999).

34. Sonne, N., Karsdal, M. A. & Henriksen, K. Mono and dual agonists of the amylin, calcitonin, and CGRP receptors and their potential in metabolic diseases. *Mol. Metab.* **46**, 101109 (2020).

35. Rodriguez Camargo, D. C. *et al.* Cloning, expression and purification of the human Islet Amyloid Polypeptide (hIAPP) from *Escherichia coli*. *Protein Expr. Purif.* **106**, 49–56 (2015).

36. Kosicka, I. *et al.* Preparation of uniformly 13C,15N-labeled recombinant human amylin for solid-state NMR investigation. *Protein Expr. Purif.* **99**, 119–130 (2014).

37. Ciaraldi, T. P., Goldberg, M., Odom, R. & Stolpe, M. In vitro effects of amylin on carbohydrate metabolism in liver cells. *Diabetes* **41**, 975–981 (1992).

38. Lopes, D. H. J. *et al.* Amyloidogenicity and Cytotoxicity of Recombinant Mature Human Islet Amyloid Polypeptide (rhIAPP)*. *J. Biol. Chem.* **279**, 42803–42810 (2004).

39. Mazor, Y., Gilead, S., Benhar, I. & Gazit, E. Identification and Characterization of a Novel Molecular-recognition and Self-assembly Domain within the Islet Amyloid Polypeptide. *J. Mol. Biol.* **322**, 1013–1024 (2002).

40. Yamashita, S. *et al.* Escherichia coli-based production of recombinant ovine angiotensinogen and its characterization as a renin substrate. *BMC Biotechnol.* **16**, 33 (2016).

41. Palanikumar, I. *et al.* Heterologous expression, purification, and functional characterization of recombinant ovine angiotensinogen in the methylotrophic yeast Pichia pastoris. *Biotechnol. Prog.* **35**, e2866 (2019).

42. CellMosaic. Angiotensin II (1-8) (human, horse, sheep, pig, and rat). *CellMosaic* https://cellmosaic.com/angiotensin-ii-1-8-human-horse-sheep-pig-and-rat/.

43. DailyMed - GIAPREZA- angiotensin ii injection. https://dailymed.nlm.nih.gov/dailymed/drugInfo.cfm?setid=c265d69a-3efe-4107-9a9e-e6fd3d531c48.

44. Senatore, F. *et al.* FDA Approval of Angiotensin II for the Treatment of Hypotension in Adults with Distributive Shock. *Am. J. Cardiovasc. Drugs Drugs Devices Interv.* **19**, 11–20 (2019).

45. Alam, A. *et al.* Angiotensin II: A Review of Current Literature. *J. Cardiothorac. Vasc. Anesth.* **36**, 1180–1187 (2022).

46. Tai, H.-M., Li, C.-C., Hung, C.-Y. & Yin, L.-J. Production of functional peptides with inhibition ability against angiotensin I-Converting enzyme using P. pastoris expression system. *J. Food Drug Anal.* **26**, 1097–1104 (2018).

47. Yan, L. *et al.* Analysis of Transmembrane Domains 1 and 4 of the Human Angiotensin II AT1 Receptor by Cysteine-scanning Mutagenesis*. *J. Biol. Chem.* **285**, 2284–2293 (2010).

48. PubChem. Argipressin. https://pubchem.ncbi.nlm.nih.gov/compound/644077.

49. Bovy, P. *et al.* Synthesis and biological activity of N-terminus modified [Ile8] angiotensin II analogues. *Eur. J. Med. Chem.* **25**, 589–594 (1990).

50. Penn, J. *et al.* Heterologous production of daptomycin in Streptomyces lividans. *J. Ind. Microbiol. Biotechnol.* **33**, 121–128 (2006).

51. Tzotzos, S. J. Daptomycin – A New Twist To An Old Tale. *The European Peptide Society* https://www.eurpepsoc.com/daptomycin-twist-tale/ (2016).

52. LaPlante, K. L. & Rybak, M. J. Daptomycin – a novel antibiotic against Gram-positive pathogens. *Expert Opin. Pharmacother.* **5**, 2321–2331 (2004).

53. Heidary, M. *et al.* Daptomycin. *J. Antimicrob. Chemother.* **73**, 1–11 (2018).

54. Cubicin | European Medicines Agency (EMA). https://www.ema.europa.eu/en/medicines/human/EPAR/cubicin (2018).

55. Liu, X. *et al.* De novo biosynthesis of daptomycin through module construction and assembly in Escherichia coli. *Chem. Eng. J.* **519**, 165182 (2025).

56. Eureka. Daptomycin separation and purification method. (2013).

57. Lee, D. Education - Danith Ly Group - Carnegie Mellon. http://www.chem.cmu.edu/groups/ly/edu/ (2017).

58. Silverman, J. A., Perlmutter, N. G. & Shapiro, H. M. Correlation of Daptomycin Bactericidal Activity and Membrane Depolarization in Staphylococcus aureus. *Antimicrob. Agents Chemother.* **47**, 2538–2544 (2003).

59. PubChem. Daptomycin. https://pubchem.ncbi.nlm.nih.gov/compound/21585658.

60. Germano, J. de F. *et al.* Intermittent Use of a Short-Course Glucagon-like Peptide-1 Receptor Agonist Therapy Limits Adverse Cardiac Remodeling via Parkin-dependent Mitochondrial Turnover. *Sci. Rep.* **10**, 8284 (2020).

61. Galli, M. *et al.* Cardiovascular Effects and Tolerability of GLP-1 Receptor Agonists: A Systematic Review and Meta-Analysis of 99,599 Patients. *J. Am. Coll. Cardiol.* **86**, 1805–1819 (2025).

62. Zhang, Y., Yang, D., Jia, Q., Yan, J. & An, F. The effect of glucagon-like peptide-1 receptor agonists on cardiac function and structure in patients with or without type 2 diabetes mellitus: An updated systematic review and meta-analysis. *Diabetes Obes. Metab.* **26**, 2401–2411 (2024).

63. Siddiqui, H. F. *et al.* The effect of GLP-1 receptor agonists on cardiac remodeling in heart failure patients with preserved and reduced ejection fraction: a systematic review and meta-analysis. *Heart Fail. Rev.* **30**, 991–1004 (2025).

64. DailyMed - RYBELSUS- oral semaglutide tablet. https://dailymed.nlm.nih.gov/dailymed/drugInfo.cfm?setid=27f15fac-7d98-4114-a2ec-92494a91da98.

65. Wegovy | European Medicines Agency (EMA). https://www.ema.europa.eu/en/medicines/human/EPAR/wegovy (2021).

66. Kang, M. *et al.* Display of quintuple glucagon-like peptide 1 (28–36) nonapeptide on Bacillus subtilis spore for oral administration in the treatment of type 2 diabetes. *J. Appl. Microbiol.* **130**, 314–324 (2021).

67. Zhang, Z.-Z., Yang, S.-S., Dou, H., Mao, J.-F. & Li, K.-S. Expression, purification, and C-terminal amidation of recombinant human glucagon-like peptide-1. *Protein Expr. Purif.* **36**, 292–299 (2004).

68. Qian, K. *et al.* Expression of a glucagon-like peptide-1 analogue, as a therapeutic agent for type II diabetes, with enhanced bioactivity and increased N-terminal homogeneity in Pichia pastoris. *Biotechnol. Lett.* **37**, 2229–2235 (2015).

69. PubChem. Human GLP-1-(7-36)-amide. https://pubchem.ncbi.nlm.nih.gov/compound/146156375.

70. Liu, Q. *et al.* Functional GLP-1R antibodies identified from a synthetic GPCR-focused library demonstrate potent blood glucose control. *mAbs* **13**, 1893425.

71. Bae, C. S., Yang, D. S., Lee, J. & Park, Y.-H. (ref) Improved process for production of recombinant yeast-derived monomeric human G-CSF. *Appl. Microbiol. Biotechnol.* **52**, 338–344 (1999).

72. Bashir, S., Sadaf, S., Ahmad, S. & Akhtar, M. W. (ref) Enhanced and Secretory Expression of Human Granulocyte Colony Stimulating Factor by Bacillus subtilis SCK6. *BioMed Res. Int.* **2015**, 1–9 (2015).

73. Trinh, N. T. M., Thuoc, T. L. & Thao, D. T. P. (ref) Production of recombinant human G-CSF from non-classical inclusion bodies in Escherichia coli. *Braz. J. Microbiol.* **52**, 541–546 (2021).

74. Xie, T. *et al.* (ref) A New STAT3-based Potency Assay for Human G-CSF Analog Therapeutics. *J. Pharm. Biomed. Anal.* **265**, 117056 (2025).

75. Skokowa, J. *et al.* A topological refactoring design strategy yields highly stable granulopoietic proteins. *Nat. Commun.* **13**, 2948 (2022).

76. Recombinant Human G-CSF. https://www.cellgs.com/products/recombinant-human-g-csf.html.

77. (ref) Filgrastim Facts. https://go.drugbank.com/drugs/DB00099.

78. DailyMed - NIVESTYM- filgrastim-aafi injection, solution. https://dailymed.nlm.nih.gov/dailymed/drugInfo.cfm?setid=d23ab39c-b6ec-41cf-a529-9dd2852c9d9a.

79. Gajewska, M. E., Sritharan, S. S., Santoni-Rugiu, E. & Bendstrup, E. M. (ref) Autoimmune pulmonary alveolar proteinosis in an adolescent successfully treated with inhaled rhGM-CSF (molgramostim). *Respir. Med. Case Rep.* **23**, 167–169 (2018).

80. DailyMed - LEUKINE- sargramostim injection, powder, lyophilized, for solution. https://dailymed.nlm.nih.gov/dailymed/drugInfo.cfm?setid=bb9a3820-f31a-4dd9-bef4-97094c01a398.

81. DailyMed - sargramostim injection, powder, lyophilized, for solution. https://dailymed.nlm.nih.gov/dailymed/drugInfo.cfm?setid=bb9a3820-f31a-4dd9-bef4-97094c01a398.

82. Imreplys | European Medicines Agency (EMA). https://www.ema.europa.eu/en/medicines/human/EPAR/imreplys (2025).

83. Kurzrock, R. Granulocyte-macrophage colony-stimulating factor. in *Holland-Frei Cancer Medicine. 6th edition* (BC Decker, 2003).

84. Forno, G. *et al.* N- and O-linked carbohydrates and glycosylation site occupancy in recombinant human granulocyte-macrophage colony-stimulating factor secreted by a Chinese hamster ovary cell line. *Eur. J. Biochem.* **271**, 907–919 (2004).

85. GM-CSF reporter cells | hGM-CSF cell-based assay| InvivoGen. https://www.invivogen.com/hek-blue-gmcsf.

86. Kinoshita, Y. *et al.* Expression and purification of biologically active human granulocyte-macrophage colony stimulating factor (hGM-CSF) using silkworm-baculovirus expression vector system. *Protein Expr. Purif.* **159**, 69–74 (2019).

87. Stern, A. C. & Jones, T. C. The side-effect profile of GM-CSF. *Infection* **20**, S124–S127 (1992).

88. Hong, D., Mingqiang, Z., Min, L., Changqing, C. & Jifang, M. Production of Recombinant Salmon Calcitonin by Amidation of Precursor Peptide Using Enzymatic Transacylation and Photolysis *in Vitro*. *Biochem. Biophys. Res. Commun.* **267**, 362–367 (2000).

89. Ray, M. V. L. *et al.* Production of Recombinant Salmon Calcitonin by In Vitro Amidation of an Escherichia coli Produced Precursor Peptide. *Bio/Technology* **11**, 64–70 (1993).

90. Ray, M. V. L. *et al.* Production of salmon calcitonin by direct expression of a glycine-extended precursor in *Escherichia coli*. *Protein Expr. Purif.* **26**, 249–259 (2002).

91. Nafeez Ahmed, A. *et al.* Production of biologically active recombinant salmon calcitonin in Escherichia coli and fish cell line. *Arch. Microbiol.* **207**, 44 (2025).

92. DailyMed - MIACALCIN- calcitonin salmon injection, solution. https://dailymed.nlm.nih.gov/dailymed/drugInfo.cfm?setid=26caf31f-7f7c-48a8-a1ac-35d2ceefda2b.

93. Rafferty, B., Corran, P. & Bristow, A. Multicenter collaborative study to calibrate salmon calcitonin by bioassay and high-performance liquid chromatography: establishment of the third international standard. *Bone* **29**, 84–89 (2001).

94. Camacho, P. M. *et al.* American Association of Clinical Endocrinologists/American College of Endocrinology Clinical Practice Guidelines for the Diagnosis and Treatment of Postmenopausal Osteoporosis—2020 Update. *Endocr. Pract.* **26**, 1–46 (2020).

95. PubChem. Calcitonin Salmon. https://pubchem.ncbi.nlm.nih.gov/compound/16220016.

96. Calcitonin 50 IU/1 ml solution for injection and infusion - Summary of Product Characteristics (SmPC) - (emc) | 12867. https://www.medicines.org.uk/emc/product/12867/smpc#gref.

97. McLaughlin, M. B., Awosika, A. O. & Jialal, I. Calcitonin. in *StatPearls* (StatPearls Publishing, Treasure Island (FL), 2025).

98. Feng, F. *et al.* Display of Human Proinsulin on the Bacillus subtilis Spore Surface for Oral Administration. *Curr. Microbiol.* **67**, 1–8 (2013).

99. Baeshen, N. A. *et al.* Cell factories for insulin production. *Microb. Cell Factories* **13**, 141 (2014).

100. Nilsson, J., Jonasson, P., Samuelsson, E., Ståhl, S. & Uhlén, M. Integrated production of human insulin and its C-peptide. *J. Biotechnol.* **48**, 241–250 (1996).

101. Knopp, J. L., Holder-Pearson, L. & Chase, J. G. Insulin Units and Conversion Factors: A Story of Truth, Boots, and Faster Half-Truths. *J. Diabetes Sci. Technol.* **13**, 597–600 (2018).

102. Cao, D. *et al.* Part 10: Adult and Pediatric Special Circumstances of Resuscitation: 2025 American Heart Association Guidelines for Cardiopulmonary Resuscitation and Emergency Cardiovascular Care. *Circulation* **152**, (2025).

103. Kalyani, R. R., Neumiller, J. J., Maruthur, N. M. & Wexler, D. J. Diagnosis and Treatment of Type 2 Diabetes in Adults: A Review. *JAMA* **334**, 984–1002 (2025).

104. Richter, B., Bongaerts, B. & Metzendorf, M.-I. Thermal stability and storage of human insulin. *Cochrane Database Syst. Rev.* **11**, CD015385 (2023).

105. Insulin lispro Sanofi | European Medicines Agency (EMA). https://www.ema.europa.eu/en/medicines/human/EPAR/insulin-lispro-sanofi (2018).

106. PubChem. Insulin Human. https://pubchem.ncbi.nlm.nih.gov/compound/118984375.

107. ProSpec, P. S. Insulin | Iddm2 | Protein Human Recombinant His | ProSpec. *prospecbio* https://www.prospecbio.com/insulin-2.

108. Zimmerman, R. E. & Stokell, D. J. Insulin production methods and pro-insulin constructs. (2010).

109. FDA, C. for D. E. and. Establishment and validation of an in vitro cell-based assay to assess the biological activity of insulin products. *FDA* https://www.fda.gov/drugs/regulatory-science-action/establishment-and-validation-in-vitro-cell-based-assay-assess-biological-activity-insulin-products (2024).

110. iLite® Insulin Assay Ready Cells. https://www.svarlifescience.com/products/bm3060.

111. Hahn, M. K., Giacca, A. & Pereira, S. In vivo techniques for assessment of insulin sensitivity and glucose metabolism. *J. Endocrinol.* **260**, e230308 (2024).

112. Ryaby, J. T. *et al.* Thrombin peptide TP508 stimulates cellular events leading to angiogenesis, revascularization, and repair of dermal and musculoskeletal tissues. *J. Bone Joint Surg. Am.* **88 Suppl 3**, 132–139 (2006).

113. Kantara, C. *et al.* Novel regenerative peptide TP508 mitigates radiation-induced gastrointestinal damage by activating stem cells and preserving crypt integrity. *Lab. Invest.* **95**, 1222–1233 (2015).

114. Chu, L. M. *et al.* The Effect of Thrombin Fragment (TP508) on Myocardial Ischemia Reperfusion Injury in a Model of Type 1 Diabetes. *Circulation* **122**, S162–S169 (2010).

115. Biospace. Chrysalis Receives FDA Approval to Initiate Clinical Trials with Chrysalin™ (TP508). *BioSpace* https://www.biospace.com/chrysalis-receives-fda-approval-to-initiate-clinical-trials-with-chrysalin-tp508 (2023).

116. Businesswire. Chrysalis BioTherapeutics, Inc. Announces Collaboration with NIAID to Evaluate Chrysalin® for the Treatment of Cutaneous Radiation Injury. https://www.businesswire.com/news/home/20241118314186/en/Chrysalis-BioTherapeutics-Inc.-Announces-Collaboration-with-NIAID-to-Evaluate-Chrysalin-for-the-Treatment-of-Cutaneous-Radiation-Injury (2024).

117. Olszewska-Pazdrak, B. *et al.* Nuclear Countermeasure Activity of TP508 Linked to Restoration of Endothelial Function and Acceleration of DNA Repair. *Radiat. Res.* **186**, 162–174 (2016).

118. European Medicines Agency. EU/3/18/1985 - orphan designation for treatment of acute radiation syndrome | European Medicines Agency (EMA). https://www.ema.europa.eu/en/medicines/human/orphan-designations/eu-3-18-1985 (2018).

119. PubChem. Rusalatide Acetate. https://pubchem.ncbi.nlm.nih.gov/compound/91668177.

120. Weatherspoon, J. K. & Hicks, J. L. Rusalatide acetate compositions. (2020).

121. MedChemExpress. TP508 | Thrombin Peptide | MedChemExpress. *MedchemExpress.com* https://www.medchemexpress.com/TP508.html.

122. Chyrsalis Biotherapeutics. TP508 Peptide Technology. *Chrysalis BioTherapeutics, Inc.* https://chrysbio.com/tp508-peptide-technology/ (2025).

123. Freyberg, S., Song, Y.-H., Muehlberg, F. & Alt, E. Thrombin Peptide (TP508) Promotes Adipose Tissue-Derived Stem Cell Proliferation via PI3 Kinase/Akt Pathway. *J. Vasc. Res.* **46**, 98–102 (2008).

124. Mundada, L. V. *et al.* Structure-Function Analysis of the Streptokinase Amino Terminus (Residues 1–59). *J. Biol. Chem.* **278**, 24421–24427 (2003).

125. Assiri, A. S., El-Gamal, B. A., Hafez, E. E. & Haidara, M. A. Production of recombinant streptokinase from Streptococcus pyogenes isolate and its potential for thrombolytic therapy. *Saudi Med. J.* **35**, 1482–1488 (2014).

126. Aghaeepoor, M. *et al.* Optimization and high level production of recombinant synthetic Streptokinase in E. coli using Response Surface MethodologyOptimization and High Level Production of Recombinant Synthetic Streptokinase in E. coli Using Response Surface Methodology. *Iran. J. Pharm. Res.* https://doi.org/10.22037/ijpr.2019.1100636 (2019) doi:10.22037/ijpr.2019.1100636.

127. Mahboubi, A., Sadjady, S. K., Abadi, M. M. S., Azadi, S. & Solaimanian, R. *Biological Activity Analysis of Native and Recombinant Streptokinase Using Clot Lysis and Chromogenic Substrate Assay*. 1087-1093.e125900 https://brieflands.com/journals/ijpr/articles/125900#abstract (2011) doi:10.22037/ijpr.2012.1173.

128. Adivitiya, Dagar, V. K., Devi, N. & Khasa, Y. P. High level production of active streptokinase in Pichia pastoris fed-batch culture. *Int. J. Biol. Macromol.* **83**, 50–60 (2016).

129. Molaee, N., Abtahi, H. & Mosayebi, G. Expression of Recombinant Streptokinase from Streptococcus Pyogenes and its Reaction with Infected Human and Murine Sera. *Iran. J. Basic Med. Sci.* **16**, 985–989 (2013).

130. Laha, M., Panizzi, P., Nahrendorf, M. & Bock, P. E. Engineering streptokinase for generation of active site-labeled plasminogen analogs. *Anal. Biochem.* **415**, 105–115 (2011).

131. Zhang, X.-W. *et al.* Recombinant streptokinase production by fed-batch cultivation of *Escherichia coli*. *Enzyme Microb. Technol.* **24**, 647–650 (1999).

132. Balagurunathan, B., Ramchandra, N. S. & Jayaraman, G. Enhancement of stability of recombinant streptokinase by intracellular expression and single step purification by hydrophobic interaction chromatography. *Biochem. Eng. J.* **39**, 84–90 (2008).

133. THROMBIN-JMI® (thrombin, topical, bovine origin) How Supplied/Storage and Handling | Pfizer Medical - US. https://www.pfizermedical.com/thrombin-jmi/storage-handling.

134. Raplixa | European Medicines Agency (EMA). https://www.ema.europa.eu/en/medicines/human/EPAR/raplixa (2017).

135. DailyMed - THROMBIN-JMI (thrombin, topical- bovine kit. https://dailymed.nlm.nih.gov/dailymed/drugInfo.cfm?setid=ba8fcebf-a924-488f-983d-088892761f0e.

136. Barkhordari, A. *et al.* Direct Cloning, Expression and Purification of Human Activated Thrombin in Prokaryotic System and CD Analysis Report of Produced Thrombin: Molecular Characterization of Recombinant Thrombin. *Int. J. Pept. Res. Ther.* **26**, 2661–2673 (2020).

137. Osadská, M., Boňková, H., Krahulec, J., Stuchlík, S. & Turňa, J. Optimization of expression of untagged and histidine-tagged human recombinant thrombin precursors in Escherichia coli. *Appl. Microbiol. Biotechnol.* **98**, 9259–9270 (2014).

138. Di Cera, E. Thrombin. *Mol. Aspects Med.* **29**, 203–254 (2008).

139. Garger, S. *et al.* BAY 81-8973, a full-length recombinant factor VIII: manufacturing processes and product characteristics. *Haemoph. Off. J. World Fed. Hemoph.* **23**, e67–e78 (2017).

140. PubChem. Thrombin B-Chain (147-158) (human). https://pubchem.ncbi.nlm.nih.gov/compound/90470996.

141. Ishizaki, J. *et al.* Production of recombinant human glucagon in the form of a fusion protein in Escherichia coli; recovery of glucagon by sequence-specific digestion. *Appl. Microbiol. Biotechnol.* **36**, (1992).

142. GlucaGen® Analogues and Insulins Storage & Stability | Scientific Exchange. https://www.novonordiskmedical.com/product-information/storage-and-stability/glucagen-and-insulins.html.

143. Ogluo | European Medicines Agency (EMA). https://www.ema.europa.eu/en/medicines/human/EPAR/ogluo (2021).

144. PubChem. Glucagon. https://pubchem.ncbi.nlm.nih.gov/compound/16132283.

145. Rix, I., Nexøe-Larsen, C., Bergmann, N. C., Lund, A. & Knop, F. K. Glucagon Physiology. in *Endotext* (eds Feingold, K. R. et al.) (MDText.com, Inc., South Dartmouth (MA), 2000).

146. Yoon, K.-H. *et al.* Bacterial production and structure-function validation of a recombinant glucagon peptide. *Process Biochem.* **138**, 23–32 (2024).

147. Ye, N. *et al.* Gut Delivery of Pentameric GLP-1 Using Genetically Engineered Bacillus subtilis for Diabetes and Obesity Treatment. 2025.10.07.680898 Preprint at https://doi.org/10.1101/2025.10.07.680898 (2025).

148. Wen, C., Wang, Z., Du, P., Gan, R. & Zhu, S. Secretion expression of recombinant glucagon inEscherichia coli. *Sci. China C Life Sci.* **44**, 233–240 (2001).

149. RayBio. Glucagon, Human Recombinant, His Tag. https://www.raybiotech.com/glucagon-human-recombinant-his-tag-228-12629.

150. ProSpec. Glucagon Protein Human His Tag | GCG Hormone | ProSpec. *prospecbio* https://www.prospecbio.com/glucagon_human_his.

151. Biotechne. Recombinant Human Glucagon His Protein (NBP1-72384) by Novus. https://www.bio-techne.com/p/proteins-enzymes/glucagon-recombinant-protein_nbp1-72384.

152. Wang, L.-F. *et al.* Synthesis and refolding of human tissue-type plasminogen activator in Bacillus subtilis. *Gene* **84**, 127–133 (1989).

153. Khan, M. W., Musgrave, S. C. & Jenkins, N. Production and Post-Translational Modification of Tissue Plasminogen Activator (tPA) in Namalwa Cells. in *Animal Cell*

Technology: Developments Towards the 21st Century (eds Beuvery, E. C., Griffiths, J. B. & Zeijlemaker, W. P.) 397–401 (Springer Netherlands, Dordrecht, 1995). doi:10.1007/978-94-011-0437-1_63.

154. Qiu, J., Swartz, J. R. & Georgiou, G. Expression of Active Human Tissue-Type Plasminogen Activator in Escherichia coli. *Appl. Environ. Microbiol.* **64**, 4891–4896 (1998).
155. Mohammadi, E., Seyedhosseini-Ghaheh, H., Mahnam, K., Jahanian-Najafabadi, A. & Mir Mohammad Sadeghi, H. Reteplase: Structure, Function, and Production. *Adv. Biomed. Res.* **8**, 19 (2019).
156. Gravanis, I. & Tsirka, S. E. Tissue-type plasminogen activator as a therapeutic target in stroke. *Expert Opin. Ther. Targets* **12**, 159–170 (2008).
157. Shaw, G. J., Sperling, M. & Meunier, J. M. Long-term stability of recombinant tissue plasminogen activator at -80 C. *BMC Res. Notes* **2**, 117 (2009).
158. Song, T. *et al.* Recombinant Plasminogen Activator of the Sandworm (Perinereis aibuhitensis) Expression in Escherichia coli. *Bioengineering* **11**, 1030 (2024).
159. Long, X. *et al.* Soluble expression, purification, and characterization of active recombinant human tissue plasminogen activator by auto-induction in E. coli. *BMC Biotechnol.* **15**, 13 (2015).
160. PubChem. Streptokinase. https://pubchem.ncbi.nlm.nih.gov/compound/9815560.
161. FDA Vasopressin Data Sheet. Drugs. *FDA* https://www.fda.gov/drugs (2025).
162. Bankir, L., Bichet, D. G. & Morgenthaler, N. G. Vasopressin: physiology, assessment and osmosensation. *J. Intern. Med.* **282**, 284–297 (2017).

163. Vasopressin actions in the kidney renin angiotensin system and its role in hypertension and renal disease. in *Vitamins and Hormones* vol. 113 217–238 (Academic Press, 2020).

164. Novus Biologicals. Recombinant Human V1a Vasopressin R/AVPR1A GST (N-Term) Protein. *Novus Biologicals* https://www.novusbio.com/products/v1a-vasopressin-r-avpr1a-partial-recombinant-protein_h00000552-q01.

165. Meienhofer, J., Trzeciak, A., Havran, R. T. & Walter, R. Solid-phase synthesis of [8-arginine]-vasopressin through a crystalline protected nonapeptide intermediate and biological properties of the hormone. *J. Am. Chem. Soc.* **92**, 7199–7202 (1970).

166. FDA Vasopressin Data Sheet. DailyMed - VASOSTRICT- vasopressin injection. https://dailymed.nlm.nih.gov/dailymed/drugInfo.cfm?setid=b1147beb-743e-4c62-8927-91192447f8b8.

167. Cirella, A. *et al.* Interleukin-18 in cancer immunology and immunotherapy. *Expert Opin. Ther. Targets* **27**, 1035–1042 (2023).

168. Robertson, M. J. *et al.* A Dose-Escalation Study of Recombinant Human Interleukin-18 Using Two Different Schedules of Administration in Patients with Cancer. *Clin. Cancer Res.* **14**, 3462–3469 (2008).

169. Li, X. *et al.* IL-18 binding protein (IL-18BP) as a novel radiation countermeasure after radiation exposure in mice. *Sci. Rep.* **10**, 18674 (2020).

170. Singh, V. K., Romaine, P. L. & Newman, V. L. Biologics as countermeasures for acute radiation syndrome: where are we now? *Expert Opin. Biol. Ther.* **15**, 465–471 (2015).

171. Girard-Guyonvarc'h, C., Harel, M. & Gabay, C. The Role of Interleukin 18/Interleukin 18-Binding Protein in Adult-Onset Still's Disease and Systemic Juvenile Idiopathic Arthritis. *J. Clin. Med.* **11**, 430 (2022).

172. Production of recombinant human long-acting IL-18 binding protein: inhibitory effect on ulcerative colitis in mice. *ResearchGate* https://doi.org/10.1007/s00253-023-12806-8 (2025) doi:10.1007/s00253-023-12806-8.

173. Gabay, C. *et al.* Open-label, multicentre, dose-escalating phase II clinical trial on the safety and efficacy of tadekinig alfa (IL-18BP) in adult-onset Still's disease. *Ann. Rheum. Dis.* **77**, 840–847 (2018).

174. Aizawa, Y. *et al.* Cloning and expression of interleukin-18 binding protein. *FEBS Lett.* **445**, 338–342 (1999).

175. IL-18 BP, Human - GenScript. https://www.genscript.com/protein/Z03168-IL_18_BP_Human.html?page_no=1&position_no=3&sensors=googlesearch.

176. AB2 Bio Ltd. *Multicenter, Double-Blind, Placebo-Controlled, Randomized Withdrawal Trial With Tadekinig Alfa (r-hIL-18BP) in Patients With IL-18 Driven Monogenic Autoinflammatory Conditions: NLRC4 Mutation and XIAP Deficiency*. https://clinicaltrials.gov/study/NCT03113760 (2025).

177. Novick, D. *et al.* Interleukin-18 Binding Protein: A Novel Modulator of the Th1 Cytokine Response. *Immunity* **10**, 127–136 (1999).

178. Novick, D. IL-18 and IL-18BP: A Unique Dyad in Health and Disease. *Int. J. Mol. Sci.* **25**, 13505 (2024).

179. Huard, A. *et al.* Development of anti-murine IL-18 binding protein antibodies to stimulate IL-18 bioactivity. *J. Immunol. Baltim. Md 1950* **214**, 180–191 (2025).

180. Zhang, X.-G. *et al.* Expression and Purification of Delta Sleep-Inducing Peptide Fused with Protein Transduction Domain and Human Serum Albumin in Pichia pastoris. *Protein Pept. Lett.* **24**, 668–675.

181. Oh, K. S. Expression and purification of delta sleep-inducing peptide in Escherichia coli. *ResearchGate* https://www.researchgate.net/publication/286873697_Expression_and_purification_of_delta_sleep-inducing_peptide_in_Escherichia_coli (2025).

182. Mu, X. *et al.* Pichia pastoris secreted peptides crossing the blood-brain barrier and DSIP fusion peptide efficacy in PCPA-induced insomnia mouse models. *Front. Pharmacol.* **15**, (2024).

183. Abbexa - Antibodies, Proteins, ELISA kits. *Abbexa* https://www.abbexa.com/.

184. PubChem. delta Sleep-inducing peptide, N-tyr-. https://pubchem.ncbi.nlm.nih.gov/compound/5490353.

185. Bes, F., Hofman, W., Schuur, J. & Van Boxtel, C. Effects of Delta Sleep-Inducing Peptide on Sleep of Chronic Insomniac Patients: A Double-Blind Study. *Neuropsychobiology* **26**, 193–197 (1992).

186. Pomfrett, C. J. D. *et al.* Delta sleep-inducing peptide alters bispectral index, the electroencephalogram and heart rate variability when used as an adjunct to isoflurane anaesthesia. *Eur. J. Anaesthesiol.* **26**, 128–134 (2009).

187. Phoenix Pharmaceuticals. Delta-Sleep-Inducing Peptide (DSIP). *Phoenix Pharmaceuticals, Inc.* https://phoenixpeptide.com/products/delta-sleep-inducing-peptide-dsip/.

188. Schoenenberger, G. A., Maier, P. F., Tobler, H. J., Wilson, K. & Monnier, M. The delta EEG (sleep)-inducing peptide (DSIP). *Pflüg. Arch.* **376**, 119–129 (1978).

189. Epoetin Alfa Hexal | European Medicines Agency (EMA). https://www.ema.europa.eu/en/medicines/human/EPAR/epoetin-alfa-hexal (2017).

190. Eichler-Stahlberg, A., Weisheit, W., Ruecker, O. & Heitzer, M. Strategies to facilitate transgene expression in Chlamydomonas reinhardtii. *Planta* **229**, 873–883 (2009).

191. Eprex by Janssen Data Sheet EPO Alpha. https://www.medsafe.govt.nz/.

192. DailyMed - EPOGEN- epoetin alfa solution. https://dailymed.nlm.nih.gov/dailymed/drugInfo.cfm?setid=1f2d0b28-9cc5-4523-80b8-637fdaf3f7a5.

193. PubChem Epoetin Alfa. Epoetin Alfa. https://pubchem.ncbi.nlm.nih.gov/compound/92043599.

194. Egrie, J. C. E. & Browne, J. K. B. Development and Characterization of Darbepoetin Alfa | CancerNetwork. https://www.cancernetwork.com/view/development-and-characterization-darbepoetin-alfa (2025).

195. Jelkmann, W. Physiology and Pharmacology of Erythropoietin. *Transfus. Med. Hemotherapy* **40**, 302–309 (2013).

196. Schilling, T. *How to Make Antibodies and Other Disulfide-Bonded Proteins with Bacillus Subtilis*. (University of Groningen, [Groningen], 2024). doi:10.33612/diss.998519079.

197. Adamíková, J., Antošová, M. & Polakovič, M. Chromatographic purification of recombinant human erythropoietin. *Biotechnol. Lett.* **41**, 483–493 (2019).

198. Broudy, V. C., Tait, J. F. & Powell, J. S. Recombinant human erythropoietin: Purification and analysis of carbohydrate linkage. *Arch. Biochem. Biophys.* **265**, 329–336 (1988).

199. Molnár, T., Bartošová, M., Antošová, M., Škultéty, Ľ. & Polakovič, M. Design of a three-step chromatographic process of recombinant human erythropoietin purification. *Sep. Purif. Technol.* **267**, 118673 (2021).

200. Kittur, F. S., Lalgondar, M., Hung, C.-Y., Sane, D. C. & Xie, J. C-Terminally fused affinity Strep-tag II is removed by proteolysis from recombinant human erythropoietin expressed in transgenic tobacco plants. *Plant Cell Rep.* **34**, 507–516 (2015).

201. Zhang, J. & Lan, N. Hirudin variants production by genetic engineered microbial factory. *Biotechnol. Genet. Eng. Rev.* **34**, 261–280 (2018).

202. PubChem. Lepirudin. https://pubchem.ncbi.nlm.nih.gov/compound/118856773.

203. Public Health - Public Health - European Commission. https://health.ec.europa.eu/index_en (2025).

204. Wang, Z., Böttcher, D., Bornscheuer, U. T. & Müller, C. Expression of Recombinant Hirudin in Bacteria and Yeast: A Comparative Approach. *Methods Protoc.* **8**, 89 (2025).

205. Hadadian, S. *et al.* The Effect of Signal Sequence and His-tags on the Expression and Anti-Thrombin Activity of Recombinant Hirudin. *ResearchGate* https://www.researchgate.net/publication/353526544_The_Effect_of_Signal_Sequence_and_His-tags_on_the_Expression_and_Anti-_Thrombin_Activity_of_Recombinant_Hirudin (2021).

206. Lipiäinen, T. *et al.* Formulation and Stability of Cytokine Therapeutics. *J. Pharm. Sci.* **104**, 307–326 (2015).

207. Kaye, J. A. FDA licensure of neumega® to prevent severe chemotherapy-induced thrombocytopenia. *Stem Cells* **16**, 207–223 (1998).

208. Baldo, B. A. Side Effects of Cytokines Approved for Therapy. *Drug Saf.* **37**, 921–943 (2014).

209. Center for Drug Evaluation andResearch. Drug Approvals and Databases. *FDA* https://www.fda.gov/drugs/development-approval-process-drugs/drug-approvals-and-databases (2025).

210. Pfizer Neumega Product Sheet. NEUMEGA®(oprelvekin). (2011).

211. Wilde, M. I. & Faulds, D. Oprelvekin: a review of its pharmacology and therapeutic potential in chemotherapy-induced thrombocytopenia. *BioDrugs Clin. Immunother. Biopharm. Gene Ther.* **10**, 159–171 (1998).

212. Neumega: FDA-Approved Drugs. https://www.accessdata.fda.gov/scripts/cder/daf/index.cfm (1997).

213. BPS Bioscience. Human Interleukin-11 Recombinant (Discontinued). https://bpsbioscience.com/human-interleukin-11-recombinant-90176-a (2021).

214. Yu, K.-M. *et al.* Efficient expression and isolation of recombinant human interleukin-11 (rhIL-11) in Pichia pastoris. *Protein Expr. Purif.* **146**, 69–77 (2018).

215. Amari, J. V. & Mazsaroff, I. Analysis of recombinant human interleukin-11 fusion protein derived from *Escherichia coli* lysate by combined size-exclusion and reversed-phase liquid chromatography. *J. Chromatogr. A* **729**, 113–124 (1996).

216. Tan, H., Dan, G., Gong, H. & Cao, L. Purification and characterization of recombinant truncated human interleukin-11 expressed as fusion protein in Escherichia coli. *Biotechnol. Lett.* **27**, 905–910 (2005).

217. Su, Y. *et al.* Facile production of tag-free recombinant human interleukin-11 by transforming into soluble expression in *Escherichia coli*. *Protein Expr. Purif.* **197**, 106107 (2022).

218. Yokota, H. *et al.* Quantitative in vitro bioassay for recombinant human interleukin-11. *J. AOAC Int.* **83**, 1053–1057 (2000).

219. Goldman, S. J. Preclinical biology of interleukin 11: A multifunctional hematopoietic cytokine with potent thrombopoietic activity. *Stem Cells* **13**, 462–471 (1995).

220. Lokau, J., Kespohl, B., Kirschke, S. & Garbers, C. The role of proteolysis in interleukin-11 signaling. *Biochim. Biophys. Acta BBA - Mol. Cell Res.* **1869**, 119135 (2022).

221. Singh, V. K. & Seed, T. M. Entolimod as a radiation countermeasure for acute radiation syndrome. *Drug Discov. Today* **26**, 17–30 (2021).

222. Romiplostim Injection: MedlinePlus Drug Information. https://medlineplus.gov/druginfo/meds/a609008.html (2025).

223. Nplate | European Medicines Agency (EMA). https://www.ema.europa.eu/en/medicines/human/EPAR/nplate (2018).

224. PubChem. Romiplostim. https://pubchem.ncbi.nlm.nih.gov/compound/Romiplostim.

225. Hashemzaei, M., Negahdaripour, M., Heidari, R. & Ghoshoon, M. B. Protein Expression and Purification of Romiplostim and Analysis of Its Secretory Production Using an In Silico Investigated Signal Peptide in E. Coli. *Rep. Biochem. Mol. Biol.* **12**, 27–35 (2023).

226. Kuter, D. J. Biology and Chemistry of Thrombopoietic Agents. *Semin. Hematol.* **47**, 243–248 (2010).

227. Ying, J. *et al.* Optimization of thrombopoietin mimetic peptide fusion proteins with albumin-binding domain for enhanced bioactivity and extended half-life. *Sci. Rep.* https://doi.org/10.1038/s41598-025-27504-w (2025) doi:10.1038/s41598-025-27504-w.

228. DailyMed - BIVALIRUDIN injection, powder, lyophilized, for solution. https://dailymed.nlm.nih.gov/dailymed/drugInfo.cfm?setid=c20969cb-72d4-469b-9bd8-332250d896c3.

229. Angiox | European Medicines Agency (EMA). https://www.ema.europa.eu/en/medicines/human/EPAR/angiox (2016).

230. Tovi, A. *et al.* Process for production of bivalirudin. (2013).

231. Scatena, R. Bivalirudin: a new generation antithrombotic drug. *Expert Opin. Investig. Drugs* **9**, 1119–1127 (2000).

232. PubChem. Bivalirudin. https://pubchem.ncbi.nlm.nih.gov/compound/16129704.

233. Eptifibatide Accord | European Medicines Agency (EMA). https://www.ema.europa.eu/en/medicines/human/EPAR/eptifibatide-accord (2016).

234. DailyMed - EPTIFIBATIDE injection. https://dailymed.nlm.nih.gov/dailymed/drugInfo.cfm?setid=00ad7bbe-cadd-4647-b74b-adfc9f2b5e94.

235. KANG, X., Liu, J., Ma, Y. & Yuan, J. Method for preparing eptifibatide. (2016).

236. PubChem. Eptifibatide. https://pubchem.ncbi.nlm.nih.gov/compound/448812.

237. FDA-Approved Bleeding Disorder Therapies in the US | NBDF. *National Bleeding Disorders Foundation* https://www.bleeding.org/healthcare-professionals/guidelines-on-care/products-licensed-in-the-us (2025).

238. DailyMed - Wilate- antihemophilic factor. https://dailymed.nlm.nih.gov/dailymed/drugInfo.cfm?setid=d74e59ba-bfeb-4c48-9596-da44328561a9.

239. Parti, R., Schoppmann, A., Lee, H. & Yang, L. Stability of lyophilized and reconstituted plasma/albumin-free recombinant human factor VIII (ADVATE rAHF-PFM). *Haemoph. Off. J. World Fed. Hemoph.* **11**, 492–496 (2005).

240. Mazurkiewicz-Pisarek, A., Mazurkiewicz, A., Mikiewicz, D., Baran, P. & Ciach, T. Expression of the gene encoding blood coagulation factor VIII without domain B in E. coli bacterial expression system. *BioTechnologia* **104**, 247–262 (2023).

241. Baunsgaard, D. *et al.* A comparative analysis of heterogeneity in commercially available recombinant factor VIII products. *Haemophilia* **24**, 880–887 (2018).

242. Santagostino, E. A new recombinant factor VIII: from genetics to clinical use. *Drug Des. Devel. Ther.* **8**, 2507–2515 (2014).

243. DailyMed - PROLIA- denosumab injection. https://dailymed.nlm.nih.gov/dailymed/drugInfo.cfm?setid=49e5afe9-a0c7-40c4-af9f-f287a80c5c88.

244. Rashid, M. H. Full-length recombinant antibodies from Escherichia coli: production, characterization, effector function (Fc) engineering, and clinical evaluation. *mAbs* **14**, 2111748 (2022).

245. PubChem. Denosumab. https://pubchem.ncbi.nlm.nih.gov/compound/Denosumab.

246. Schoen, P. *et al.* Anti-RANKL nanobody ALX-0141 shows sustained biomarker inhibition in a Phase I study in healthy postmenopausal Women. in *Bone Abstracts* vol. 1 (Bioscientifica, 2013).

247. Robblee, J., Collins, B. E., Kaundinya, G. & Bosques, C. J. Methods related to denosumab. (2017).
